# High-Risk Microgranular Acute Promyelocytic Leukemia with a Five-Way Complex Translocation Involving PML-RARA

**DOI:** 10.1155/2015/343854

**Published:** 2015-01-26

**Authors:** Benjamin Powers, Diane Persons, Deepthi Rao, Janet Woodroof, Tara L. Lin

**Affiliations:** ^1^Division of Hematology/Oncology, Department of Internal Medicine, University of Kansas School of Medicine, 3901 Rainbow Boulevard, Kansas City, KS 66210, USA; ^2^Department of Pathology and Laboratory Medicine, University of Kansas School of Medicine, 3901 Rainbow Boulevard, Kansas City, KS 66210, USA

## Abstract

Acute promyelocytic leukemia (APL) is classically characterized by chromosomal translocation (15;17), resulting in the PML-RARA fusion protein leading to disease. Here, we present a case of a 50-year-old man who presented with signs and symptoms of acute leukemia with concern for APL. Therapy was immediately initiated with all-*trans* retinoic acid. The morphology of his leukemic blasts was consistent with the hypogranular variant of APL. Subsequent FISH and cytogenetic analysis revealed a unique translocation involving five chromosomal regions: 9q34, 17q21, 15q24, 12q13, and 15q26.1. Molecular testing demonstrated PML/RARA fusion transcripts. Treatment with conventional chemotherapy was added and he went into a complete remission. Given his elevated white blood cell count at presentation, intrathecal chemotherapy for central nervous system prophylaxis was also given. The patient remains on maintenance therapy and remains in remission. This is the first such report of a 5-way chromosomal translocation leading to APL. Similar to APL with chromosomal translocations other than classical t(15;17) which result in the typical PML-RARA fusion, our patient responded promptly to an ATRA-containing regimen and remains in complete remission.

## 1. Introduction

Acute promyelocytic leukemia (APL) accounts for about 5% of acute myeloid leukemias. Chromosomal translocation t(15;17)(q22;q21) is the hallmark of APL, resulting in the fusion of the promyelocytic gene (PML) on chromosome 15 to the retinoic acid alpha receptor gene (RARA) on chromosome 17. Variant translocations which also result in this PML-RARA fusion have been reported in approximately 9% of cases, with similar sensitivity to all-*trans* retinoic acid (ATRA) as classical APL [1]. The majority of these variants are still associated with formation of a PML-RARA fusion gene, but by a more indirect manner. Complex translocations are defined as rearrangements involving at least 3 chromosomes and account for 1-2% of all APL cases [1]. Many variant breakpoints have been described previously [2–4]. A translocation involving 12q13 in APL has been described once previously, in a patient with an eight-way variant translocation [1]. Here we present a case with a unique five-way translocation involving PML-RARA, leading to ATRA-sensitive APL.

## 2. Case Presentation

A 53-year-old male farmer presented to an outside hospital with complaints of easy bruising and increasing fatigue. He reported fevers up to 39°C and chills two days prior to evaluation. He denied weight loss, night sweats, epistaxis, or gingival bleeding but noted dyspnea with exertion. On exam, he was in no distress and afebrile. He had scattered ecchymoses without lymphadenopathy or hepatosplenomegaly. He had a leukocytosis of 82 K/UL, hemoglobin 14.6 g/L, and platelet count of 36 K/UL, and labs consistent with disseminated intravascular coagulopathy (INR 1.5, fibrinogen 121 mg/dL). He did not have hyperuricemia, renal dysfunction, or electrolyte disturbances suggestive of tumor lysis syndrome. He was admitted for further work-up, which included a bone marrow aspiration and biopsy performed on hospital day number 1. Due to concerns for acute myeloid leukemia, he was transferred to our institution late on a Friday evening on hospital day number 2.

The past medical history was significant for coronary artery disease status after drug-eluting stent placed 9 months earlier, hypertension, and some arthritic pains of his shoulder. The patient lived at home with his girlfriend. His 15-year-old daughter stayed with him every other week and his son lived within a 10-mile radius. He reported no occupational or radiation exposures. There was no family history of hematologic malignancies, and he had no siblings.

**Figure 1:**
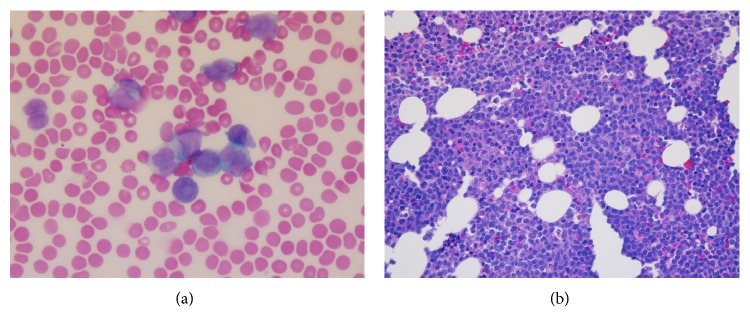
(a) Peripheral smear revealing blasts that contained bilobed nuclei with very fine granules (100x). (b) Bone marrow biopsy showing a hypercellular marrow with 64% blasts and decreased trilineage hematopoiesis (40x).

Upon transfer, the peripheral smear revealed nearly 100% blasts with no Auer rods or coarse granularity. Many of the blasts contained dumbbell-shaped nuclei with very fine granules concerning the hypogranular variant of APL (Figure 1(a)). He was immediately started on ATRA and dexamethasone given his elevated WBC count, as well as prophylactic allopurinol and antibiotics. He was given cryoglobulin to maintain fibrinogen >150 mg/dL and platelet transfusions to goal 50 K/UL.

**Figure 2:**
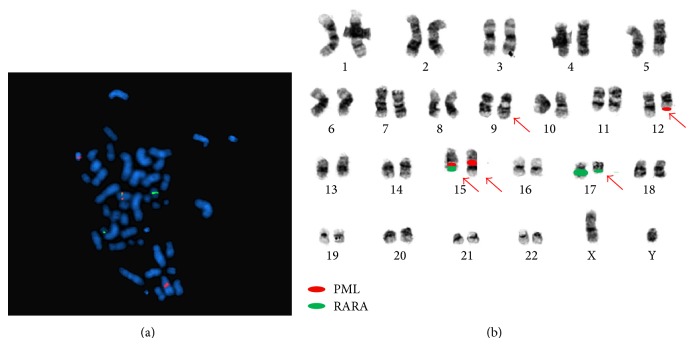
(a) Metaphase FISH: dual-color dual-fusion FISH probe has one fusion, two orange (PML) and two green (RARA) signals on representative metaphase nuclei. (b) Illustration of positions of FISH probes based on several DAPI (as in (a)) and reverse DAPI (not shown) metaphase studies confirming the complex translocation: t(9;17;15;12;15)(q34;q21;q24;q13;q26.1).

The bone marrow aspirate and biopsy showed a hypercellular marrow (90–100% cellular with 64% blasts) and decreased trilineage hematopoiesis (Figure 1(b)). Butyrate esterase stain was negative for monocytic differentiation. Flow cytometry showed blasts comprising 92% of total cells, positive for dim CD2 (aberrant), dim CD4 (aberrant), CD13, CD33, CD34, dim CD38, CD56 (aberrant), CD64, CD117, partial HLA-DR, and myeloperoxidase. Fluorescence in situ hybridization (FISH) using probes for the PML (15q24.1) and RARA (17q21.1) regions showed a signal pattern consistent with PML/RARA rearrangement and a complex translocation involving t(15;17) in all 200 nuclei assessed (Figure 2(a)). FISH using a chromosome 17 probe and ABL1 probe confirmed that a portion of 9q was present on the derivative chromosome 17 (not shown). Conventional cytogenetics revealed that 18 of 20 metaphases had a complex translocation involving five chromosomal regions: 9q34, 17q21, 15q24, 12q13, and 15q26.1. The final karyotype was 46,XY,t(9;17;15;12;15)(q34;q21;q24;q13;q26.1)[18]/46,XY[2] (Figure 2(b)).

He was deemed high-risk due to initial leukocyte count and treated definitively with ATRA and idarubicin. On the second day of ATRA plus anthracycline, he became hypoxic and short of air, with concern for ATRA differentiation syndrome. ATRA was held for two days and symptoms resolved, after which ATRA was restarted. He had no further complications.

A month later, repeat bone marrow analysis confirmed complete remission by morphology. Cytogenetics confirmed resolution of the complex translocation and RT-PCR was unable to detect any PML-RARA fusion transcripts. Because of his initial high-risk presentation, he received prophylactic intrathecal chemotherapy with induction and consolidation per Tallman and Altman's recommendation [5]. He continues in complete morphologic and molecular remission and is completing maintenance chemotherapy per the PETHEMA protocol [6].

## 3. Discussion

Similar to our patient, APL without the typical t(15;17) but with the common PML-RARA fusion is usually sensitive to ATRA induction therapy. However, ATRA resistance is seen with APL resulting from t(11;17)(q23;q21), when RARA is fused to the PML-linked zinc finger (PLZF). This entity is characterized by a retinoid-independent binding of corepressors and therefore, a lack of differentiation of promyelocytes when ATRA is attempted [1]. This specific translocation does not necessarily affect prognosis, as case reports suggest complete responses in patients who received conventional chemotherapy as well [1]. Whether it is from classical t(15;17) or more complex cytogenetic abnormalities, the presence of typical PML-RARA gene rearrangement appears to be most predictive of a response to ATRA.

Our patient's case is unique given this five-way translocation with 46,XY,t(9;17;15;12;15)(q34;q21;q24;q13;q26.1) resulting in PML-RARA fusion and APL. Similar to other variant APL chromosomal translocations resulting in PML-RARA fusion, his APL was sensitive to ATRA therapy and he continues to do well clinically.
